# Supplementary material for: Sustainability effects of motor control stabilisation exercises on pain and function in chronic nonspecific low back pain patients: A systematic review with meta-analysis and meta-regression
Source: PLoS One. 2020 Jan 15;15(1):e0227423. doi: 10.1371/journal.pone.0227423 (PMC6961919; doi:10.1371/journal.pone.0227423)
Supplement: Attachment — Submitted filename: PONE-D-19-29553_reviewer.pdf [file pone.0227423.s002.pdf]

PLOS ONE

Sustainability effects of motor control stabilisation exercises on pain and function in chronic nonspecific low back pain patients: A systematic review with meta-analysis and meta-regression

--Manuscript Draft--

Manuscript Number:	PONE-D-19-29553
Article Type:	Research Article
Full Title:	Sustainability effects of motor control stabilisation exercises on pain and function in chronic nonspecific low back pain patients: A systematic review with meta-analysis and meta-regression
Short Title:	Sustainability effects of motor control stabilisation exercises in chronic nonspecific low back pain patients
Corresponding Author:	Daniel Niederer<br>Goethe-Universitat Frankfurt am Main<br>Frankfurt, GERMANY
Keywords:	sensorimotor; stabilisation; core-stability; lumbalgia; backache; long-term effects
Abstract:	Study Design: Systematic review with meta-analysis and meta-regression<br><br>Background and Objectives: We systematically reviewed and delineated the existing evidence on sustainability effects of motor control exercises on pain intensity and disability in chronic low back pain patients when compared with an inactive or passive control group or with other exercises. Secondary aims were to reveal whether moderating factors like the time after training cessation, the study quality, and the training characteristics affect the potential sustainability effects.<br>Methods: Relevant scientific databases (Medline, Web of Knowledge, Cochrane) were screened. Eligibility criteria for selecting studies: All RCTs und CTs on chronic (≥ 12/13 weeks) nonspecific low back pain, written in English or German and adopting a longitudinal core-specific/stabilizing sensorimotor control exercise intervention with at least one pain intensity and disability outcome assessment at follow-up (sustainability).<br><br>Results and Conclusions: From the 3,415 studies that were initially retrieved, 10 (2 CTs & 8 RCTs) on N=1081 patients were included in the review and analyses. Low to moderate quality evidence shows a sustainable positive effect of motor control exercise on pain (SMD=-.46, Z=2.9, p<.001) and disability (SMD=-.44, Z=2.5, p<.001) in low back pain patients when compared to any control. The subgroups' effects are less conclusive and no clear direction of the sustainability effect at short versus mid versus long-term, of the type of the comparator, or of the dose of the training is given. Low quality studies overestimated the effect of motor control exercises.
Order of Authors:	Daniel Niederer
	Juliane Mueller
Additional Information:	
Question	Response
**Financial Disclosure**<br><br>Enter a financial disclosure statement that describes the sources of funding for the work included in this submission. Review the submission guidelines for detailed requirements. View published research articles from *PLOS ONE* for specific examples.	The authors received no specific funding for this work.

Powered by Editorial Manager® and ProduXion Manager® from Aries Systems Corporation

This statement is required for submission and **will appear in the published article** if the submission is accepted. Please make sure it is accurate.<br><br>**Unfunded studies**<br>Enter: *The author(s) received no specific funding for this work.*<br><br>**Funded studies**<br>Enter a statement with the following details:<br>• Initials of the authors who received each award<br>• Grant numbers awarded to each author<br>• The full name of each funder<br>• URL of each funder website<br>• Did the sponsors or funders play any role in the study design, data collection and analysis, decision to publish, or preparation of the manuscript?<br>• **NO** - Include this sentence at the end of your statement: *The funders had no role in study design, data collection and analysis, decision to publish, or preparation of the manuscript.*<br>• **YES** - Specify the role(s) played.<br><br>* typeset	
**Competing Interests**<br><br>Use the instructions below to enter a competing interest statement for this submission. On behalf of all authors, disclose any competing interests that could be perceived to bias this work—acknowledging all financial support and any other relevant financial or non-financial competing interests.<br><br>This statement **will appear in the published article** if the submission is accepted. Please make sure it is accurate. View published research articles from *PLOS ONE* for specific examples.	The authors have declared that no competing interests exist

Powered by Editorial Manager® and ProduXion Manager® from Aries Systems Corporation

**NO authors have competing interests**<br><br>Enter: *The authors have declared that no competing interests exist.*<br><br>**Authors with competing interests**<br><br>Enter competing interest details beginning with this statement:<br><br>*I have read the journal's policy and the authors of this manuscript have the following competing interests: [insert competing interests here]*<br><br>* typeset	
**Ethics Statement**<br><br>Enter an ethics statement for this submission. This statement is required if the study involved:<br><br>• Human participants<br>• Human specimens or tissue<br>• Vertebrate animals or cephalopods<br>• Vertebrate embryos or tissues<br>• Field research<br><br>Write "N/A" if the submission does not require an ethics statement.<br><br>General guidance is provided below. Consult the submission guidelines for detailed instructions. **Make sure that all information entered here is included in the Methods section of the manuscript.**	N/A

**Format for specific study types**<br><br>**Human Subject Research (involving human participants and/or tissue)**<br>• Give the name of the institutional review board or ethics committee that approved the study<br>• Include the approval number and/or a statement indicating approval of this research<br>• Indicate the form of consent obtained (written/oral) or the reason that consent was not obtained (e.g. the data were analyzed anonymously)<br><br>**Animal Research (involving vertebrate animals, embryos or tissues)**<br>• Provide the name of the Institutional Animal Care and Use Committee (IACUC) or other relevant ethics board that reviewed the study protocol, and indicate whether they approved this research or granted a formal waiver of ethical approval<br>• Include an approval number if one was obtained<br>• If the study involved *non-human primates*, add *additional details* about animal welfare and steps taken to ameliorate suffering<br>• If anesthesia, euthanasia, or any kind of animal sacrifice is part of the study, include briefly which substances and/or methods were applied<br><br>**Field Research**<br><br>Include the following details if this study involves the collection of plant, animal, or other materials from a natural setting:<br>• Field permit number<br>• Name of the institution or relevant body that granted permission	
**Data Availability**<br><br>Authors are required to make all data underlying the findings described fully available, without restriction, and from the time of publication. PLOS allows rare exceptions to address legal and ethical concerns. See the PLOS Data Policy and FAQ for detailed information.	Yes - all data are fully available without restriction

Powered by Editorial Manager® and ProduXion Manager® from Aries Systems Corporation

A Data Availability Statement describing where the data can be found is required at submission. Your answers to this question constitute the Data Availability Statement and **will be published in the article**, if accepted.<br><br>**Important:** Stating 'data available on request from the author' is not sufficient. If your data are only available upon request, select 'No' for the first question and explain your exceptional situation in the text box.<br><br>Do the authors confirm that all data underlying the findings described in their manuscript are fully available without restriction?	
**Describe where the data may be found in full sentences. If you are copying our sample text, replace any instances of XXX with the appropriate details.**<br><br>• If the data are **held or will be held in a public repository**, include URLs, accession numbers or DOIs. If this information will only be available after acceptance, indicate this by ticking the box below. For example: *All XXX files are available from the XXX database (accession number(s) XXX, XXX.).*<br>• If the data are all contained **within the manuscript and/or Supporting Information files**, enter the following: *All relevant data are within the manuscript and its Supporting Information files.*<br>• If neither of these applies but you are able to provide **details of access elsewhere**, with or without limitations, please do so. For example:<br><br>*Data cannot be shared publicly because of [XXX]. Data are available from the XXX Institutional Data Access / Ethics Committee (contact via XXX) for researchers who meet the criteria for access to confidential data.*<br><br>*The data underlying the results presented in the study are available from (include the name of the third party*	Sytematic review ith meta-analyses. All data are displayed within the manuscript.

Powered by Editorial Manager® and ProduXion Manager® from Aries Systems Corporation

and contact information or URL).<br>• This text is appropriate if the data are owned by a third party and authors do not have permission to share the data.<br><br>* typeset	
Additional data availability information:	

Powered by Editorial Manager® and ProduXion Manager® from Aries Systems Corporation

Click here to download Manuscript Plos One Long-term effects of MCE in LBP Syst-Review_MA.docx

Sustainability effects and long-term dose-response relationships of motor control exercises on pain and function in chronic nonspecific low back pain patients: A systematic review with meta-analysis and meta-regression

Niederer, Daniel[a] & Mueller, Juliane[b]

[a]Department of Sports Medicine, Institute of Sports Sciences, Goethe University Frankfurt, Frankfurt am Main, Germany

[b]Department of Computer Science / Therapy Sciences, Professorship for Physiotherapy: Exercise Science and Applied Biomechanics, Trier University of Applied Sciences, Trier, Germany

Daniel Niederer
Department of Sports Medicine, Goethe University Frankfurt,
Ginnheimer Landstraße 39; 60487 Frankfurt am Main, Germany
+49 (0)69 798 245 81; niederer@em.uni-frankfurt.de, ORCID: 0000-0002-7690-5418

Conflict of interest: none

Acknowledgements: none

Sustainability effects of motor control stabilisation exercises on pain and function in chronic nonspecific low back pain patients: A systematic review with meta-analysis and meta-regression

Introduction

A multitude of hypothesized and confirmed risk factors for both the onset and chronification of nonspecific low back pain is available in the literature. Beyond psychological and social factors [1], neuromuscular factors (i.e. deficits or impairments) are particularly named [2,3]. Neuromuscular impairments may be successfully treated. Target-oriented interventions to improve neuromuscular deficits, in particular sensorimotor training, is one of the most established therapy form in low back pain treatment [4,5]. The effects of sensorimotor movement therapies on pain and disability decreases, as well as on function improvements, are highlighted in numerous meta-analyses on chronic nonspecific low back pain. Motor-control exercises [5] and Pilates-based stabilization exercises [6] have been shown to be superior to minimal intervention. When compared to other active interventions, motor control and Pilates-based stabilisation exercises provide similar outcomes to other forms of exercises [5,6]. In contrast, Wang et al. [7] found in their meta-analysis that core-stability exercises are more effective than general exercises. Likewise, back pain-oriented stabilization exercises provided greater effects than general exercise [8]. In general, strength/resistance and coordination/stabilisation exercise programs seem to be superior to other interventions in the treatment of chronic low back pain [4]. Taken together, sensorimotor training is one of the most effective active regimens for chronic low back pain treatment. Beyond these short or intermediate-term pre-to-post-intervention effects, motor control exercise is likewise superior to inactivity or minimal intervention in the long-term [5]. Compared to other forms of active exercise, stabilisation and core stability exercise was found to be no more effective than in the long term [9][7].

The lack of uniform definitions hinders researchers and practitioners in interpreting conflicting evidence and adopting adequate measures in terms of sensorimotor training. Motor control, sensorimotor, perturbation, neuromuscular, core stability, stabilization, Pilates-based and instability trainings are often used to describe sensorimotor training principles. Musculoskeletal control by afferent sensory, in particular proprioceptive, input, central nervous system integration and optimal motor control to assure functional dynamic joint stability during perturbative situations, are key components of all the training forms described above [10]. Studies using these appropriate muscle recruitment patterns and timing key components as the adequate motor answer on perturbations of a (stable) system as trainings principles may thus be pooled in analyses on motor control stabilisation exercises. As different definitions and/or definitions with overlaps to non-dynamic motor control situations are often summarized under the term motor control (where, classically, pre-education on deep trunk muscles activation and/or the control of deep muscles activation during exercising is done), the pooled effects of (not only but also) long-term effects may have been over- or underestimated. Furthermore, most of the reviews reported intermediate or long-term effects by aggregating effect sizes with a certain (homogeneous) duration after the randomization. Due to the different intervention durations adopted in the different studies included, long-term effects of the interventions (where the effect are assessed during or immediately after therapy) are thus mixed/pooled with short, intermediate, and long-term sustainability effects (where the effect was assessed after a certain time after exercise cessation). It is thus often unclear as to whether 1) reported long-term effects of motor control stabilisation exercises are based on interventions adopting a rigorous definition of sensorimotor exercises, and 2) if the effects are really based on sustainability effects or rather long-term interventions. Likewise, determining the optimal dose for maximal treatment success (response) is still a matter of debate [11,12].

Against the research deficit highlighted above, the research questions of the present systematic review with meta-analysis and meta-regression are: (1) do motor control stabilisation exercises lead to a sustainable improvement of pain intensity and disability in chronic nonspecific low back pain patients compared to an inactive or passive control group or compared to other exercises; and (2) to what

extent do moderating factors like the duration of the time after training cessation, the study quality, and the training characteristics affect the potential sustainability effects?

Methods

Study design

This secondary data analysis was conducted as a systematic review with meta-analysis and meta-regression. The Preferred Reporting Items for Systematic Reviews and Meta-Analysis (PRISMA) guidelines were followed when conducting and reporting this review.

Inclusion & exclusion criteria

The inclusion and exclusion criteria were defined with respect to PICO (population, intervention, control/comparator, outcome. The criteria for both the participants and studies are displayed in Table 1.

Literature research

The literature research was performed using the peer review-based databases PubMed (Medline), Web of Knowledge, and the Cochrane Library. Potentially relevant articles were searched adopting the following Boolean search syntax (example for the PubMed search):

(stabili* OR sensorimotor OR “motor control” OR neuromuscular OR perturbation) AND (exercise OR training OR therapy OR intervention OR treatment) AND ("low back pain" OR lumbalgia OR "lower back pain" OR dorsalgia OR backache OR lumbago OR LBP OR “back pain”).

An initial exploratory electronic database search was conducted by two independent reviewers (JM and DN) to define the final search terms. Both reviewers independently conducted the main research afterwards. The herewith identified studies were screened for eligibility using 1) titles and 2) abstracts. The remaining full texts were assessed to ascertain whether they are fulfilling the inclusion and not fulfilling the exclusion criteria. Consensus was used to address any disparities; a third reviewer (N.N.) was asked, if necessary, to address any disparities. After study retrieval, additional

studies were identified by manually searching through the reference list (cross-referencing) of the selected articles.

Data extraction

The included studies were screened for common effect estimators (for pain intensity and disability). Standard mean differences between intervention and comparator effect sizes were calculated based on mean and standard deviation values for the respective scale. Data for the sustainability effects in the short term (≥ 4 weeks ≤ 3 months), medium term (> 3 and ≤ 12months) and long term (> 12 months) after training cessation were collected. All data of interest (descriptive, PICO, interventional details, study quality and risk of bias) were retrieved from the individual study data. For that purpose, a data extraction form, designed for this review, was u[illegible]ed. One researcher recorded all the pertinent data from the included articles and the other author independently reviewed the extracted data for its relevance, accuracy and comprehensiveness. Consensus was used to address any disparities; a third reviewer (N.N.) was asked, if necessary, to address any disparities. Authors of studies included in this review who have not reported sufficient details in the published manuscript were personally addressed per e-mail for the provision of further data. Effect estimators (pain intensity and disability) were primarily calculated using the visual analogue scale (VAS) or the numeric rating scale (NRS) or sum score inherent of the scale/assessment tool (0-10 or 0-24 or 0-100), as the calculation of the standard mean differences is scale-independent. For such data, only the direction (lower values mean less pain, less disability) was normalized. For scale-dependent calculations (inverse weighting), z-transformed (0-10) variab[illegible]es were used. Missing standard deviations for the differences were imputed according to the procedure described in Follmann et al. [13].

Study quality assessment

The methodological quality of all controlled trials included was assessed using the PEDro scale (11 criteria). The PEDro scale is a valid and reliable tool to assess the methodological quality of controlled

studies [14]. Each criterion was rated as 1 (definitely yes) or 0 (unclear or no); potential disagreements were discussed between the two authors and then resolved.

Risk of Bias within studies/outcomes

The two review authors (JM and DN) independently rated the risk of bias of the included studies, using the Cochrane Collaboration's tool [15]. Following the Cochrane recommendations, bias was rated outcome specific and not study specific (Cochrane Handbook Version 5.1.0, Chapter 8.7). The outcomes were graded for risk of bias in each of the following domains: sequence generation, allocation concealment, blinding (participants, personnel, and outcome assessment), incomplete outcome data, selective outcome reporting, and other sources of bias. Each item was rated as "high risk", "low risk", or "unclear risk" of bias. Again, any disagreements were discussed between the raters. If a decision could not be reached after discussion, a third reviewer (N.N.) was included to resolve any conflicts. If applicable. The outcomes' bias were reported pooled for studies.

Measures of treatment effects – main effects

The Review Manager 5.3 (RevMan, Version 5.3, Copenhagen: The Nordic Cochrane Centre, The Cochrane Collaboration, 2014) was used for data analyses of the main effects. Standardised means differences and sample sizes were used for data pooling. A random-effects meta-analysis model for continuous outcomes was chosen. For variance description, 95 % confidence intervals were calculated; data were displayed using Forrest-plots. To test for overall effects, Z-statistics at a 5 % alpha-error-probability level were calculated for: 1. Overall (main) effects and 2. Quantitative subgroup analyses. For the overall effect calculation, each intervention group effects was calculated in contrast to the comparator/control group. In studies with more than two MCE arms, more than one effect estimator contributes to the main calculation. If more than one sustainability timepoint was assessed, the mid-term sustainability effect was selected for the main analysis. For the quantitative subgroup calculations, analyses were performed separately for 2a. sensitivity of time (short-term, mid-term, and long-term sustainability), and 2b. sensitivity of comparator (inactive or passive vs. motor control

stabilisation exercises (MCE) and other exercises vs. MCE). For variance description of the subgroup analyses, 90 % confidence intervals were calculated; data were displayed using Forrest-plots. To test for overall effects, Z-statistics at a 5 % alpha-error level were calculated.

Measures of treatment effects – assessment of heterogeneity

Clinical heterogeneity between the study results in effect measures was assessed using I^2-statistic. An I-squared value greater than 50 % is indicative for substantial heterogeneity [15].

Measures of treatment effects – Sensitivity meta-regression for dose-response analyses

To counteract the considerable heterogeneity, sensitivity meta-regressions for dose-response analyses and the impact of study quality and risk of bias were conducted. A syntax for SPSS (IBM SPSS 23; IBM, USA) was used (David B. Wilson; Meta-Analysis Modified Weighted Multiple Regression; MATRIX procedure Version 2005.05.23). Inverse variance weighted regression models with random intercepts (random effect model, fixed slopes model) with the dependent variables pain and disability effects (simple pre-post Cohen's ds) and the independent variables intervention duration [weeks], intervention frequency [number of trainings/weeks], intervention [ratio of the sustainability time / training time], intervention total dose [minutes], and study quality PEDro sum score [points]. Homogeneity analysis (Q and p-values) and meta-regression partial coefficients B (95 % confidence intervals and p-values) were calculated.

Risk of bias across studies

The calculation of the risk of publication bias across all studies is indicated by using funnel plots/graphs (Simmonds 2015).

Effect estimators' level of evidence

Quality of evidence revealed by the main and subgroup meta-analyses were graded using the tool established by the GRADE working group [16]. Quality evidence was categorized as "very low", "low" "moderate", or "high" (plus interim values).

Results

Study selection

The database search was completed in 10/2018. Figure 1 displays the research procedure and the flow of the study selection and inclusion.

Study characteristics and individual studies' results

Ten (10) studies were included in the qualitative and quantitative sustainability analyses. Their characteristics and main results are displayed in Table 2. For each of the studies included, methodological aspects, participants' characteristics, and key results are displayed. Overall, 1,081 participants with nonspecific chronic low back pain were included.

Two of the studies are controlled trials (CT) [17][18], while the other eight adopted a randomized controlled design (RCT) [19] [20] [21] [22] [23] [24] [25] [26]. Main inclusion criterion was (chronic) nonspecific low back pain ≥ 6 weeks (1x, [24]), ≥ 8 weeks (1x, [25]), ≥12 weeks (3 x, [19], [20][21]), 24 weeks (1x, [17]). The baseline pain (VAS, 0-10 points) ranged from 2.9±0.8 [19] to 6.5±2.1[21]. The effect sizes (Cohens d, MCE only) for the sustainability measures ranged from .27 [18] to 2.6 [26] (pain intensity) and, for disability, from .17 [19] to 1.9 [24].

Study quality and risk of bias within studies (outcomes)

Both the study quality and risk of bias ratings are displayed in table 3. Overall study quality was 5/11 to 9/11 points, with a mean of 6.8. As the outcomes were assessed using self-reported questions within the same questionnaires, the risk of bias was reported accumulated per study and not per outcome.

Main effect estimates

The main effect size estimates of the overall sustainability (4 to 44 weeks after training cessation) effects of motor control stabilisation exercise in comparison to inactive control, passive treatment or other exercises for the outcomes pain intensity and disability are displayed in Figure 2.

Low to moderate quality evidence indicates that MCE has a larger overall sustainability effect on pain intensity and disability than a passive, inactive or other exercise comparator.

Grouped effect estimates

Figures 3 to 8 show the main effect estimates results as pooled forest plots, separated for sustainability duration after training cessation (short-term: Figure 3 and Figure 4, mid-term: Figure 5 and Figure 6, and long-term: Figure 7 and Figure 8), for the type comparator (passive or inactive control, Figure 3, Figure 4, Figure 5; other exercise, Figure 4, Figure 6, Figure 8), and for the outcomes pain intensity (figures 3-8, parts –A-) versus disability (figures 3-8, parts –B-).

Low quality evidence indicates that MCE has no larger short-term sustainability effect on pain intensity than a passive or inactive comparator. Low quality evidence indicates that MCE has a larger short-term sustainability effect on disability than a passive or inactive comparator.

Low quality evidence indicates that MCE has no larger short-term sustainability effect on pain intensity and disability than other exercises.

Low quality evidence indicates that MCE has no larger mid-term sustainability effect on pain intensity than a passive or inactive comparator. Moderate quality evidence indicates that MCE has no larger mid-term sustainability effect on disability than a passive or inactive comparator.

Low to moderate quality evidence indicates that MCE has no larger mid-term sustainability effect on pain intensity than other exercises. Low to moderate quality evidence indicates that MCE has no larger mid-term sustainability effect on disability than other exercises.

Moderate quality evidence indicates that MCE has no larger long-term sustainability effect on pain intensity than a passive or inactive comparator. Moderate quality evidence indicates that MCE has no larger long-term sustainability effect on disability than a passive or inactive comparator.

Low to moderate quality evidence indicates that MCE has a larger long-term sustainability effect on pain intensity than other exercises. Low to moderate quality evidence indicates that MCE has no larger long-term sustainability effect on disability than other exercises.

Individual studies: training characteristics

Table 4 summarizes the individual studies' training characteristics. All interventions and the respective comparators are described. The motor control stabilisation exercises are named MCE: [21–23] , core stability exercises: [19], stabilization: [20,24,25,27] sensorimotor [17], sling training [18], and Pilates-based exerc[illegible] [26]. Six out of the ten studies adopted an eight-week intervention, and the mean training time was 53 minutes. Training frequency ranged from 1 [18] to 12 [21] times per week.

Sensitivity Meta-Regressions on training characteristics

The results of the five meta-regressions as sensitivity analyses are highlighted in Table 5. The training duration, frequency, total trainings dose and training-to-sustainability ratio showed no impact on the effect size of the primary outcome pain.

The PEDro sum score was negatively associated with the effect size, a study with a score-decrease of 1 point shows an increase in the effect size of .24. Figure 9 illustrates this association.

Risk of bias across studies

The risk of bias across studies (publication bias) is, by means of a funnel plot, highlighted in Figure 10. It reveals an unclear but rather low risk of publication bias.

Discussion

Summary of the evidence

Low to moderate quality evidence indicates that MCE has a larger overall sustainability effect on pain intensity and disability reduction than a passive, inactive or other exercise comparator.

Subgroup sensitivity analyses revealed with low quality evidence that, in the short-term sustainability, MCE has no larger short-term effect on pain intensity than a passive or inactive comparator or than other exercises. Low quality evidence indicates that MCE has a larger short-term sustainability effect on disability, but not on pain intensity, than a passive or inactive comparator. In the mid-term sustainability, low to moderate quality evidence indicates that MCE has no larger effect on pain intensity or disability than a passive or inactive comparator or than other exercises. Moderate quality evidence further indicates that MCE has no larger long-term sustainability effect on disability than a passive or inactive comparator but a larger long-term sustainability effect on pain intensity than other exercises. Low to moderate quality evidence indicates that MCE has no larger long-term sustainability effect on disability than other exercises or passive or inactive treatments.

The subsequent meta-regression demonstrated that the training duration, frequency, total trainings dose and training-to-sustainability ratio has no impact on the effect size of the primary outcome pain. The PEDro sum score was negatively associated with the effect size and studies with lower quality may overestimate the (sustainability) effects of MCE on pain intensity and disability reduction.

Small overall effects for a larger effect of MCE than other controls/exercises are seen; the subgroup analyses revealed inconsistent results. Here, MCE is at least equivalent to other forms of exercise.

Comparison with other evidence

To compare our findings with other published evidence, the limitations of MCE training definition and the mix of long-term and sustainability effects highlighted in the introduction must be consid-

ered. First, most available evidence focusses on long-term effects (in duration after the randomization) and not on sustainability. Thus, a mix of sustainability effects and effects directly assessed after the training cessation are mixed. Second, not all evidence-based analyses used key components of appropriate muscle recruitment patterns and timing as the adequate motor answer to perturbations of a (stable) system as inclusion criterion for the trainings.

A recent meta-analysis on core-stability trainings in low back pain patients found no follow-up differences in pain reduction between core stability exercise and general exercise [7]. The findings are based on a limited number of studies. A comparable amount of analyses on numerous RCTs adopting stabilization training demonstrated heterogeneous results which are comparable to ours [9]. The authors found a systematic benefit of stabilization exercises on pain intensity when compared with any alternative treatment or control at an intermediate follow-up of 3-12 months and at a long-term follow-up of >12 months. In contrast, they found strong evidence that stabilization exercises are not more effective than any other form of active exercise in the long-term. In the meta-analysis on MCE [5], the authors concluded that there is high-quality evidence for no clinically important standardized difference of MCE for pain intensity (when compared to other exercises) or disability (when compared to minimal intervention) at intermediate and long-term follow-ups. When compared to minimal intervention, MCE was found to be in favour of a clinically important effect of pain intensity changes at medium and long-term follow-ups [5].

Practical relevance

Overall, MCE seems to be slightly more sustainable or at least equivalent to other exercises and slightly more sustainable than passive or inactive treatments in terms of pain intensity and disability reduction. Although, derived from the quality of evidence of the findings, no grade A recommendation can be provided, but MCE seems to be both effective and safe in the treatment of low back pain. Further, none of the other types of exercise was elicited to be more effective. Therapy should, of course, always be patient-centred and focussed on the individual context and preferences of the patient [28]. Based on the individual patient’s preferences, the findings of our review, and proper dose-

response relations plus training characteristics, the effects of MCE interventions will most likely be increased in the future.

A suggested underlying mechanism for the general exercise effect in low back pain is mostly seen in the analgesic effect of exercise. Exercise releases beta-endorphins, both spinal and supraspinal, by activating μ-opioid receptors [29]. Following that, an acute sensible decrease in pain is felt. In the long term, exercise and, in particular, sensorimotor motor control training may increase the functional capacity of all involved tissues, leading to a protection against neuromuscular-deficient motor patterns [30].

Limitations at study and outcome level

A common limitation in exercise trials is the limited possibility to blind the participants. This limitation is increased by the subjective assessment of pain and pain–related function. We showed that a lower study quality is associated with larger effect sizes (MCE groups only). The (overall) effect of the MCE may thus be overestimated. This finding is most likely attributed to the lack of participant and study personnel blinding. The finding of an overestimation of the effect in lower quality studies have been demonstrated in other disease therapies, like depression [31]. More high-quality evidence is thus needed to prove our findings.

Limitations at review level

The funnel plot analysis revealed an unclear but rather low risk of publication bias within our review. As the risk is nevertheless unclear, and the findings of the main analyses were heterogeneous, future study potentially affects the main findings towards positive effects of MCE compared to other interventions (most likely), no difference between MCE and other exercises (likely) or larger effect in other exercises (unlikely).

The transfer of our results into practice may be limited against the proper definition of the studies' populations and therapy aims. Although all studies name long-term, follow-up or sustainability effects in chronic low back pain patients as the aim of the intervention, it remains unclear as to

whether chronic, chronic-recurrent or even subacute participants were included. In chronic-recurrent and subacute patients, the effects are rather sustainability of the therapy but effects of tertiary prevention. Only limited evidence is available if tertiary prevention is effective when adopting exercise in general, and MCE in particular [32,33]. To provide further evidence, running RCTs should differ in their reports between tertiary (recurrence) prevention, long-term effects and sustainability [34].

Perspective

We found low to moderate quality evidence for a sustainable positive effect of motor control stabilisation exercise on pain and disability in low back pain patients when compared to any control. The subgroups effects are less clear, and no clear direction of short vs. mid vs. long-term, nor of the type or dose of the comparator, is given. Low-quality studies overestimate the effects of motor control stabilisation exercises. Further high-quality studies are needed to prove or adopt our findings.

Acknowledgements: none

Author contributions: Idea: DN and JM, literature research and rating: DN and JM; data extraction and meta-caculations: DN and JM; Manuscript writing and approval: DN and JM

Competing interests and funding support: none

References

1. Hartvigsen J, Lings S, Leboeuf-Yde C, Bakketeig L. Psychosocial factors at work in relation to low back pain and consequences of low back pain; a systematic, critical review of prospective cohort studies. Occup Environ Med. 2004; 61: e2.
2. Borghuis J, Hof AL, Lemmink KAPM. The importance of sensory-motor control in providing core stability: implications for measurement and training. Sports Med. 2008; 38: 893–916. doi: 10.2165/00007256-200838110-00002.
3. Brown SHM, McGill SM. The intrinsic stiffness of the in vivo lumbar spine in response to quick releases: implications for reflexive requirements. Journal of Electromyography and Kinesiology. 2009; 19: 727–736. doi: 10.1016/j.jelekin.2008.04.009.

4. Searle A, Spink M, Ho A, Chuter V. Exercise interventions for the treatment of chronic low back pain: a systematic review and meta-analysis of randomised controlled trials. Clin Rehabil. 2015; 29: 1155–1167. doi: 10.1177/0269215515570379.

5. Saragiotto BT, Maher CG, Yamato TP, Costa LOP, Menezes Costa LC, Ostelo RWJG, et al. Motor control exercise for chronic non-specific low-back pain. Cochrane Database Syst Rev. 2016: CD012004. doi: 10.1002/14651858.CD012004.

6. Lim ECW, Poh RLC, Low AY, Wong WP. Effects of Pilates-based exercises on pain and disability in individuals with persistent nonspecific low back pain: a systematic review with meta-analysis. JOURNAL OF ORTHOPAEDIC & SPORTS PHYSICAL THERAPY. 2011; 41: 70–80. doi: 10.2519/jospt.2011.3393.

7. Wang X-Q, Zheng J-J, Yu Z-W, Bi X, Lou S-J, Liu J, et al. A meta-analysis of core stability exercise versus general exercise for chronic low back pain. PLoS One. 2012; 7: e52082. doi: 10.1371/journal.pone.0052082.

8. Gomes-Neto M, Lopes JM, Conceição CS, Araujo A, Brasileiro A, Sousa C, et al. Stabilization exercise compared to general exercises or manual therapy for the management of low back pain: A systematic review and meta-analysis. Phys Ther Sport. 2017; 23: 136–142. doi: 10.1016/j.ptsp.2016.08.004.

9. Smith BE, Littlewood C, May S. An update of stabilisation exercises for low back pain: a systematic review with meta-analysis. BMC Musculoskelet Disord. 2014; 15: 416. doi: 10.1186/1471-2474-15-416.

10. Riemann BL, Lephart SM. The Sensorimotor System, Part I: The Physiologic Basis of Functional Joint Stability. J Athl Train. 2002; 37: 71–79.

11. Hicks GE, Fritz JM, Delitto A, McGill SM. Preliminary development of a clinical prediction rule for determining which patients with low back pain will respond to a stabilization exercise program. Arch Phys Med Rehabil. 2005; 86: 1753–1762. doi: 10.1016/j.apmr.2005.03.033.

12. Choi BK, Verbeek JH, Tam WW-S, Jiang JY. Exercises for prevention of recurrences of low-back pain. Cochrane Database Syst Rev. 2010: CD006555. doi: 10.1002/14651858.CD006555.pub2.

13. Follmann D, Elliott P, Suh I, Cutler J. Variance imputation for overviews of clinical trials with continuous response. Journal of Clinical Epidemiology. 1992; 45: 769–773. doi: 10.1016/0895-4356(92)90054-Q.

14. Morton NA de. The PEDro scale is a valid measure of the methodological quality of clinical trials: a demographic study. Aust J Physiother. 2009; 55: 129–133.

15. Higgins JPT, Altman DG, Gøtzsche PC, Jüni P, Moher D, Oxman AD, et al. The Cochrane Collaboration's tool for assessing risk of bias in randomised trials. BMJ. 2011; 343: d5928. doi: 10.1136/bmj.d5928.

16. Atkins D, Best D, Briss PA, Eccles M, Falck-Ytter Y, Flottorp S, et al. Grading quality of evidence and strength of recommendations. BMJ. 2004; 328: 1490. doi: 10.1136/bmj.328.7454.1490.

17. Giesche F, Streicher H, Maiwald M, Wagner P. [Inpatient multimodal pain therapy. Additive value of neuromuscular core stability exercises for chronic back pain]. Schmerz. 2017; 31: 115–122. doi: 10.1007/s00482-016-0178-6.

18. Unsgaard-Tøndel M, Fladmark AM, Salvesen Ø, Vasseljen O, Unsgaard-Tondel M, Salvesen O. Motor control exercises, sling exercises, and general exercises for patients with chronic low back pain. A randomized controlled trial with 1-year follow-up. Phys Ther. 2010; 90: 1426–1440. doi: 10.2522/ptj.20090421.

19. Bae C-R, Jin Y, Yoon B-C, Kim N-H, Park K-W, Lee S-H. Effects of assisted sit-up exercise compared to core stabilization exercise on patients with non-specific low back pain. A randomized controlled trial. J Back Musculoskelet Rehabil. 2018; 31: 871–880. doi: 10.3233/BMR-170997.

20. Critchley DJ, Ratcliffe J, Noonan S, Jones RH, Hurley MV. Effectiveness and cost-effectiveness of three types of physiotherapy used to reduce chronic low back pain disability. A pragmatic randomized trial with economic evaluation. Spine (Phila Pa 1976). 2007; 32: 1474–1481. doi: 10.1097/BRS.0b013e318067dc26.

21. Ferreira ML, Ferreira PH, Latimer J, Herbert RD, Hodges PW, Jennings MD, et al. Comparison of general exercise, motor control exercise and spinal manipulative therapy for chronic low back pain. A randomized trial. Pain. 2007; 131: 31–37. doi: 10.1016/j.pain.2006.12.008.

22. Macedo LG, Latimer J, Maher CG, Hodges PW, McAuley JH, Nicholas MK, et al. Effect of Motor Control Exercises Versus Graded Activity in Patients With Chronic Nonspecific Low Back Pain. A Randomized Controlled Trial. Phys Ther. 2012; 92: 363–377. doi: 10.2522/ptj.20110290.

23. Marshall PWM, Kennedy S, Brooks C, Lonsdale C. Pilates exercise or stationary cycling for chronic nonspecific low back pain. Does it matter? A randomized controlled trial with 6-month follow-up. Spine (Phila Pa 1976). 2013; 38: E952-9. doi: 10.1097/BRS.0b013e318297c1e5.

24. Rasmussen-Barr E, Nilsson-Wikmar L, Arvidsson I. Stabilizing training compared with manual treatment in sub-acute and chronic low-back pain. MANUAL THERAPY. 2003; 8: 233–241.

25. Rasmussen-Barr E, Ang B, Arvidsson I, Nilsson-Wikmar L, Äng B. Graded Exercise for Recurrent Low-Back Pain A Randomized, Controlled Trial With 6-, 12-, and 36-Month Follow-ups. Spine (Phila Pa 1976). 2009; 34: 221–228. doi: 10.1097/BRS.0b013e318191e7cb.

26. Kofotolis N, Kellis E, Vlachopoulos SP, Gouitas I, Theodorakis Y. Effects of Pilates and trunk strengthening exercises on health-related quality of life in women with chronic low back pain. J Back Musculoskelet Rehabil. 2016; 29: 649–659. doi: 10.3233/BMR-160665.

27. Gutknecht M, Mannig A, Waldvogel A, Wand BM, Luomajoki H. The effect of motor control and tactile acuity training on patients with non-specific low back pain and movement control impairment. J Bodyw Mov Ther. 2015; 19: 722–731. doi: 10.1016/j.jbmt.2014.12.003.

28. Lin I, Wiles L, Waller R, Goucke R, Nagree Y, Gibberd M, et al. What does best practice care for musculoskeletal pain look like? Eleven consistent recommendations from high-quality clinical practice guidelines: systematic review. Br J Sports Med. 2019. doi: 10.1136/bjsports-2018-099878.

29. Sharan D, Rajkumar JS, Mohandoss M, Ranganathan R. Myofascial low back pain treatment. Curr Pain Headache Rep. 2014; 18: 449. doi: 10.1007/s11916-014-0449-9.

30. Egan M, Seeger D, Schöps P. Physiotherapie und physikalische Therapie in der Schmerzmedizin. Schmerz. 2015; 29: 562–568. doi: 10.1007/s00482-015-0043-z.

31. Cuijpers P, van Straten A, Bohlmeijer E, Hollon SD, Andersson G. The effects of psychotherapy for adult depression are overestimated: a meta-analysis of study quality and effect size. Psychol Med. 2010; 40: 211–223. doi: 10.1017/S0033291709006114.

32. Bell JA, Burnett A. Exercise for the primary, secondary and tertiary prevention of low back pain in the workplace: a systematic review. J Occup Rehabil. 2009; 19: 8–24. doi: 10.1007/s10926-009-9164-5.

33. Niederer D, Vogt L, Banzer W. Physical activity, training and exercise in the prevention of low back pain: a focus review with special emphasis on motor control. Dtsch Z Sportmed. 2018; 2018: 262–266. doi: 10.5960/dzsm.2018.321.

34. Niederer D, Vogt L, Wippert P-M, Puschmann A-K, Pfeifer A-C, Schiltenwolf M, et al. Medicine in spine exercise (MiSpEx) for nonspecific low back pain patients: study protocol for a multicentre, single-blind randomized controlled trial. Trials. 2016; 17: 507. doi: 10.1186/s13063-016-1645-1.

Table 1: Inclusion and exclusion criteria for both the studies and the participants.

Criterion	Inclusion	Exclusion
Study design	Controlled	acute/immediate effects/responses<br>Case studies<br>Case-control, cohort studies<br>Reviews
Population	Adults<br>Chronic non-specific<br>(12 < weeks) low back pain patients	Children, adolescents <18yrs of age
Intervention	motor control<br>core-specific sensorimotor / neuromuscular / sensorimotor / perturbation / core stability<br>stabilization / stabilization exercises/training	Static (non-dynamic) (motor control) exercises
Control/Comparator	Active or Passive	
Outcome	At least one measure of pain (e.g., VAS, NRS, Korff) and/or disability (e.g., ODI, RMDQ, KORFF)	
Follow-up length	> 3 weeks after training cessation	
Other	Publication or e-pub before 1st October 2018<br>Language: German & English<br>Full-text availability	

Table 2: Study characteristics (left columns) and individual studies' results (right columns). For each of the studies included, methodological aspects, participants' characteristics, and key results are displayed. RCT, randomized controlled trial, CT, controlled trial; MCE, motor control stabilisation exercise, Ctrl, control or comparison group; CLBP, chronic low back pain; N, number; f, female; m, male; SD, standard deviation; Mx, measurement visit number, VAS, visual analogue scale; NRS, numeric rating scale; ODI, Owestry disability index, RMDQ, Roland Morris disability questionnaire.

First Author, year	Citation	Design, Arms	Main inclusion criterion lbp	N (Total, MCE, C, C2, ...)	Age (Mean± SD) years	Sex (f/m)	Baseline-pain (Scale, MW, SD if not stated otherwise)	Measurement time points total (N: weeks (if not, stated otherwise) after Baseline)	Primary outcome pain, name, Cohens d, (M0-M1, M0-M2, ...)	Primary outcome disability name, Cohens d, , (M0-M1, M0-M2, ...)
Bae, 2018	[19]	RCT, 2<br><br>MCE<br><br>Ctrl	CLBP ≥ 12 weeks	36<br><br>18<br><br>18	 <br>32.7±6.1<br><br>32.4±11	18/20	VAS (0-10)<br><br>2.9±0.8<br><br>3.0±1.3	4:<br>4<br>8<br>16	VAS (0-10)<br>1<br>1.25<br>1.75	ODI<br>.19<br>.17<br>.24
Critchley, 2007	[20]	RCT, 3<br><br>MCE<br><br>Ctrl 1<br>Ctrl 2	CLBP ≥ 12weeks	212<br><br>72<br><br>71<br>69	 <br>44±13<br><br>45±12<br>44±12	133/89	NRS (0-100), mean, 95%CI<br><br>67, 61-73<br><br>60, 54-66<br>59, 52-65	4:<br>6 months<br>12 months<br>18 months	NRS (0-10)<br>.7<br>.6<br>.9	RMDQ<br>1.0<br>.7<br>.8
Ferreira 2007	[21]	RCT, 3<br><br>MCE<br><br>Ctrl 1<br>Ctrl 2	CLBP ≥ 12 weeks	240<br><br>80<br><br>80<br>80	 <br>51.9±15.3<br><br>54.8±15.3<br>54.0±14.4	165/75	VAS (0-10)<br><br>6.3±2.0<br><br>6.5±2.1<br>6.2±2.0	4:<br>8<br>24<br>48	VAS (0-10)<br>.9<br>1<br>.7	RMDQ<br>1.2<br>1.1<br>.98
Giesche 2017	[17]	CT, 2<br><br>MCE<br><br>Ctrl	CLBP ≥24 weeks	48<br><br>25<br><br>23	 <br>56.5±11.3<br><br>60.1±12.2	31/17	NRS (0-10)<br><br>4.6±2.0<br><br>4.9±2.0	4:<br>2<br>3<br>8	NRS (0-10)<br>.1<br>.55<br>.65	ODI<br><br>.3<br>.34

Kofotolis, 2016	[26]	RCT, 3<br>MCE<br>Ctrl 1<br>Ctrl 2	CLBP ≥12 weeks	101<br>37<br>36<br>28	41.2±8.5<br>42.7±6.1<br>39.1±8.7	101/0	SF-36 pain<br>38.51±12.62<br>36.93±15.5<br>39.4±14.5	5:<br>4<br>8<br>12<br>20	SF-36 pain (0-100)<br>1.9<br>3,2<br>2,9	RMDQ<br>.75<br>1.2<br>1.1
Macedo, 2012	[22]	RCT, 2<br>MCE<br>Ctrl	CLBP<br>≥ 12 weeks	158<br>76<br>82	48.7±13.7<br>49.6±16.3	102/56	NRS (0-10)<br>6.1±2.1<br>6.1±1.9	4:<br>8<br>6 months<br>12 months	NRS (0-10)<br>.95<br>.95<br>1.1	RMDQ<br>.8<br>.7<br>.8
Marshall, 2013	[23]	RCT,2<br>MCE<br>Ctrl	Recurrent LBP ≥ 12 weeks	64<br>32<br>32	36.2 ± 8.2<br>36.2 ± 6.2	40/24	VAS (0-10)<br>3.6 ± 2.1<br>4.5 ± 2.5	3:<br>8<br>6 months	VAS (0-10)<br>.9<br>.76	ODI<br>.93<br>.93
Rasmussen-Barr, 2003	[24]	RCT, 2<br>MCE<br>Ctrl	LBP sub-acute, chronic or recurrent ≥ 6 weeks	42<br>22<br>20	39± 12<br>37± 10	12, 35	VAS (0-100), median 25th /75th<br>33 (27/49)<br>32 (21/49)	4:<br>6<br>3 months<br>12 months	VAS (0-100)<br>1.6<br>1.3	ODI<br>1.9<br>1.8
Rasmussen-Barr, Eva, 2009	[25]	RCT, 2<br>MCE<br>Ctrl	LBP ≥ 8 weeks	71<br>36<br>35	37± 10<br>40± 12	35, 36	VAS (0-100), VAS (0-100), median 25th /75th<br>32 (18/75)<br>38 (23/62)	5:<br>8<br>6 months<br>12 months<br>36 months	VAS (0-100),<br>.8<br>.9	OSD<br>.9<br>1.4
Unsgaard-Tondel, 2010	[18]	CT, 3<br>MCE<br>Ctrl 1<br>Ctrl 2	CLPB	109<br>36<br>36<br>37	41± 12<br>43± 10<br>36± 10	33, 76	NRS (0-10)<br>3.3 ± 1.3<br>3.6 ± 1.7<br>3.3 ± 1.9	3:<br>8<br>1 year	NRS (0-10)<br>.37<br>.27	ODI<br>.9<br>N.A.

	PEDro											**Sum PEDro**	Random sequence generation	Allocation conceal-ment	Perfor-mance bias	Detec-tion bias	Attrition bias	Reporting bias	Other bias
Number / Item	1	2	3	4	5	6	7	8	9	10	11								
Bae, 2018	1	1	0	1	0	0	0	1	1	1	1	**6**	unknown	low	high	high	low	low	low
Critchley, 2007	1	1	1	1	0	0	1	0	1	1	1	**7**	low	low	high	low	high	low	un-known
Ferreira 2007	1	1	1	1	0	1	1	1	1	1	1	**9**	low	low	high	low	low	low	low
Giesche 2017	1	0	0	1	1	0	0	0	1	1	1	**5**	high	high	high	high	high	unknown	low
Kofotolis, 2016	1	1	0	1	0	0	0	0	1	1	1	**5**	unknown	low	high	high	high	low	low
Macedo, 2012	1	1	1	1	0	0	1	1	1	1	1	**8**	low	low	high	low	low	low	low
Marshall, 2013	1	1	1	1	1	0	1	1	1	1	1	**9**	unknown	low	high	low	low	low	un-known
Rasmussen-Barr, 2003	1	1	0	1	0	0	0	1	0	1	1	**5**	unknown	low	high	high	un-known	low	un-known
Rasmussen-Barr, 2009	1	1	1	1	0	0	0	1	1	1	1	**7**	low	low	high	high	low	low	low
Unsgaard-Tondel, 2010	1	1	1	1	0	0	0	1	1	1	1	**7**	low	low	high	high	low	low	low

Table 3: Study quality and risk of bias. PEDro-scale-items: 1) eligibility criteria were specified, 2) participants were randomly allocated to groups, 3) allocation was concealed, 4) the groups were similar at baseline regarding the most important prognostic indicators, 5) there was blinding of all participants, 6) there was blinding of all therapists who administered the therapy, 7) there was blinding of all assessors who measured at least one key outcome, 8) measures of at least one key outcome were obtained from more than 85% of the subjects initially allocated to groups, 9) all subjects for whom outcome measures were available received the treatment or control condition as allocated or, where this was not the case, data for at least one key outcome was analysed by “intention to treat”, 10) the results of between-group statistical comparisons are reported for at least one key outcome, 11) the study provides both point measures and measures of variability for at least one key outcome.

Table 4: Individual studies' training characteristics. All interventions and the respective comparators are described. MCE, motor control stabilisation exercise; N.A., not applicable.

First author, year	Type MCE Intervention (MCE, CSE, Stabili, …)	Exercises (N): (Names)	Type comparator(s)	Training period (weeks)	Training Frequency (sessions per week)	Training duration (minutes per session)	Sets (number per exercise)	Repetitions (per set per exercise)	Rest (between sets per exercise; between exercises in seconds)
Bae, 2018	CSE	6: Abdominal drawing-in in 4-point kneeling and supine position, Opposite upper and lower extremity lift in quadruped position, Straight leg raise exercise in prone position, Supine lower extremity extender in supine position, Straight leg raise exercise in supine position, Horizontal side-support exercise in side lying position	Assisted sit-up exercise	4	3	30	N.A.	N.A.	N.A.
Critchley, 2007	Spinal Stabil	N.A.: individual transversus abdominis and lumbar multifidus muscle training followed by group exercises that challenged spinal stability. Exercises were tailored to assessment findings and progressed within participants' ability to maintain a stable and minimally painful spine. The exercise program aimed to improve trunk muscle motor control	Physio, Pain Management	N.A.	8	90	N.A.	N.A.	N.A.
Ferreira 2007	MCE	N.A.: Improving function of specific trunk muscles thought to control inter-segmental movement of the spine, including transversus abdominis, multifidus, the diaphragm and pelvic floor muscles (Richardson)	General exercise, Spinal manipulation therapy	8	12	N.A..	N.A.	N.A.	N.A.
Giesche 2017	Sensorimotor Stabili in add to MMST	N.A.: Exercises in lying, sitting and standing positions	MMST	2	7	60	N.A.	N.A.	N.A.

Kofotolis, 2016	Pilates	16: Roll down, mermaid, spine stretching, pelvic curl, criss-cross, double leg stretch, hundreds, double knee folds, table top, swimming, swan, cat stretch, child's pose, hips stretch	General strengthening/stabilisation exercise, control	8	3	60	2 (until week 4), then 3	15 (week 1-2), 20 (w 3-4), 15 (5-6), 20 (7-8)	2
Macedo, 2012	MCE	N.A.: Varying interindividual	General Graded Activity	8	2 (first 4 weeks), 1 (rest)	60	1	10	N.A.
Marshall, 2013	MCE & Pilates	8: Whole body stretching; Skilled abdominal contractions and postural training; Side lying trunk; Prone lying trunk; Hip-specific exercises; Upper and lower limb; Full body exercises; Whole body stretching	Stretching and cycling	8	3	55	N.A.	N.A.	N.A.
Rasmussen-Barr, 2003	Stabil	6-8: motor control, supine crooked-lying, four-point kneeling, prone, sitting and standing	Manual therapy	6	1 supervised; 7 home-based	45 supervised, 15	3	15	N.A.
Rasmussen-Barr, Eva, 2009	Graded Stabil	6-8: N.A.	30-minute walk every day	8	1 supervised; 7 home-based	45 supervised, 15 self-admin	3	15	N.A.
Unsgaard-Tondel, 2010	Sling Training	N.A.: Sling training	Low-load MCE (feedback) and General exercise	8	1	40	N.A.	N.A.	N.A.

442

Table 5: Outcomes of the sensitivity meta-regressions. For each single analysis, effect sizes, number of included effect sizes, homogeneity, the regression coefficient B, its confidence interval (CI) and the corresponding p-value are displayed.

Model (independent variable)	Mean effect size	N effect sizes in-cluded	Homo-geneity Q	B	95 % CI	p-value
Intervention: Duration [weeks]	1.01	8	2.1	-.09	-.22, .03	.15
Intervention: Frequency [$N_{Train-ings}$/weeks]	1.00	8	.0001	.0007	-.11, .11	.99
Intervention: Ratio sustaina-bility:training	1.2	15	1.3	-.04	-.11, .03	.25
Intervention: total dose [minutes]	1.0	8	.87	-.0004	-.001, .0004	.35
Study quality: Pedro [points]	1.12	15	6.1	-.24	-.43, -.05	.014

Figure captions

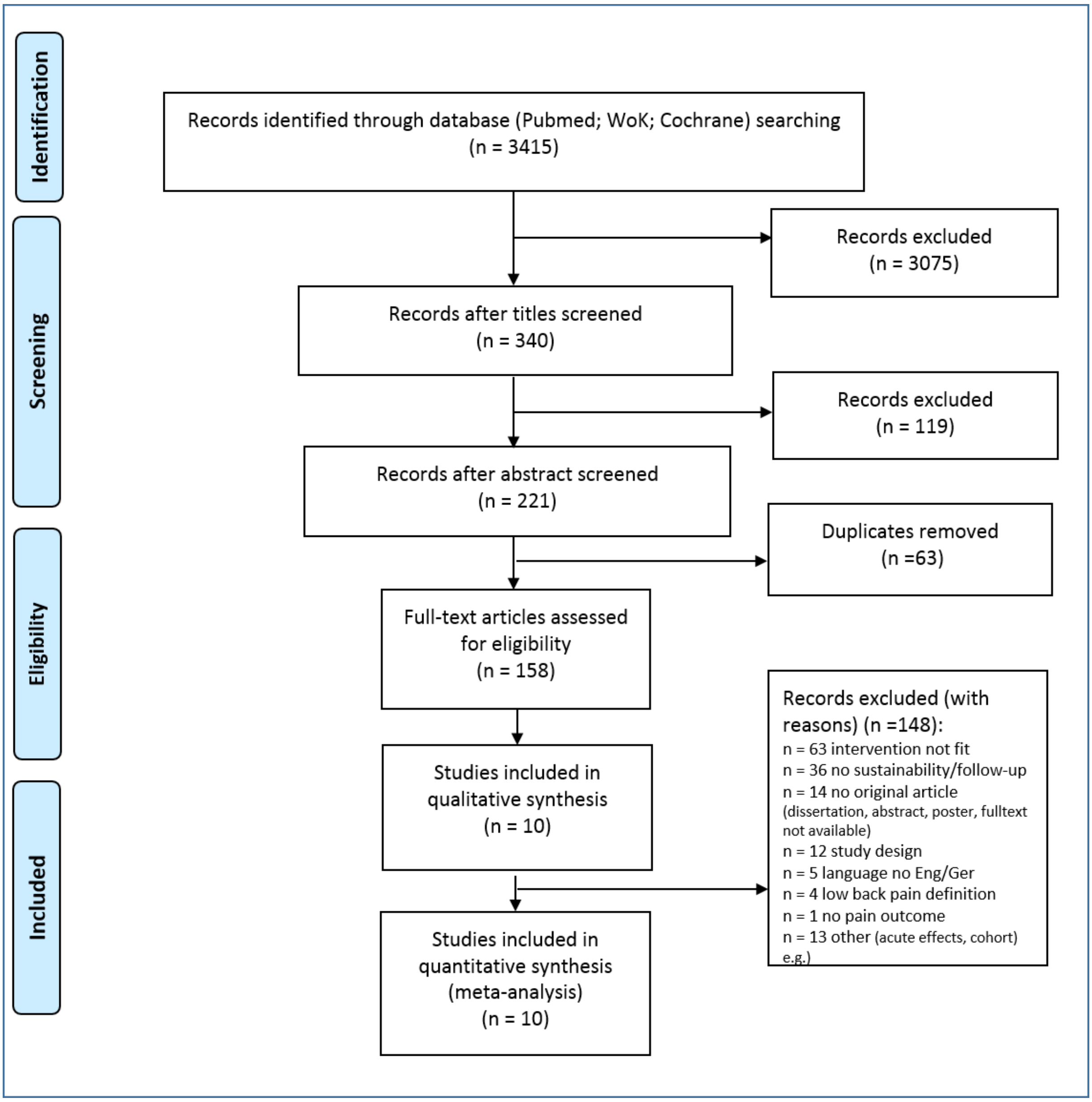

Figure 1: Research, selection and synthesis of included studies. n, number; Eng, English, Ger, German; WoK, Web of Knowledge.

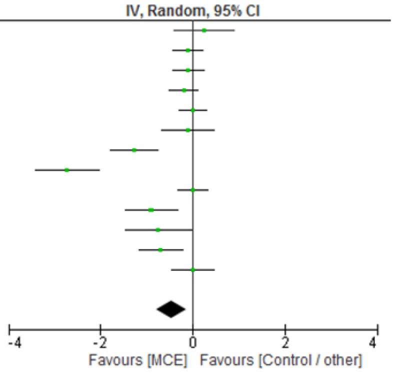

MCE vs. Inactive, passive measures, or other exercise

- A - Pain intensity

Study or Subgroup	MCE Mean	MCE SD	MCE Total	Comparator Mean	Comparator SD	Comparator Total	Weight	Std. Mean Difference IV, Random, 95% CI
Bae 2018	1.9	1.2	18	1.6	1.2	18	6.7%	0.24 [-0.41, 0.90]
Critchley 2007	39	30	72	42	28	69	8.5%	-0.10 [-0.43, 0.23]
Critchley 2007	39	30	72	42	33	71	8.5%	-0.09 [-0.42, 0.23]
Ferreira 2007	4.3	2.6	80	4.8	2.6	80	8.6%	-0.19 [-0.50, 0.12]
Ferreira 2007	4.3	2.6	80	4.3	2.6	80	8.6%	0.00 [-0.31, 0.31]
Giesche 2017	2.5	2	25	2.7	2	23	7.2%	-0.10 [-0.67, 0.47]
Kofotolis 2016	24	17	37	43	12	36	7.6%	-1.27 [-1.78, -0.77]
Kofotolis 2016	24	17	37	63	9	28	6.5%	-2.73 [-3.41, -2.04]
Macedo 2012	4.1	2.5	74	4.1	2.7	81	8.6%	0.00 [-0.32, 0.32]
Marshall 2013	1.7	2	29	3.7	2.4	23	7.2%	-0.90 [-1.48, -0.33]
Rasmussen-Barr 2003	14	12	17	27	21	16	6.4%	-0.75 [-1.46, -0.04]
Rasmussen-Barr 2009	18	15	36	32	24	35	7.7%	-0.69 [-1.17, -0.21]
Unsgaard-Tøndel 2010	2.7	2.2	36	2.7	2	37	7.8%	0.00 [-0.46, 0.46]
**Total (95% CI)**			**613**			**597**	**100.0%**	**-0.46 [-0.78, -0.14]**

Heterogeneity: Tau² = 0.28; Chi² = 85.34, df = 12 (P < 0.00001); I² = 86%
Test for overall effect: Z = 2.85 (P = 0.004)

- B - Disability

Study or Subgroup	MCE Mean	MCE SD	MCE Total	Comparator Mean	Comparator SD	Comparator Total	Weight	Std. Mean Difference IV, Random, 95% CI
Bae 2018	11.2	9.9	18	7.4	7.7	18	7.3%	0.42 [-0.24, 1.08]
Critchley 2007	7	6.1	72	6.2	6.8	69	9.1%	0.12 [-0.21, 0.45]
Critchley 2007	7	6.1	72	8	6.9	71	9.1%	-0.15 [-0.48, 0.18]
Ferreira 2007	8.4	6.4	80	7.7	6.2	80	9.2%	0.11 [-0.20, 0.42]
Ferreira 2007	8.4	6.4	80	10	7	80	9.2%	-0.24 [-0.55, 0.07]
Giesche 2017	37	15	25	42	13	23	7.9%	-0.35 [-0.92, 0.22]
Kofotolis 2016	4.1	1.9	37	11	3.9	28	7.5%	-2.33 [-2.97, -1.69]
Kofotolis 2016	4.1	1.9	37	8	2.4	36	8.0%	-1.79 [-2.33, -1.24]
Macedo 2012	8	7.1	74	8.6	6.8	81	9.2%	-0.09 [-0.40, 0.23]
Marshall 2013	15	11	29	18	12	23	8.0%	-0.26 [-0.81, 0.29]
Rasmussen-Barr 2003	8.3	6.3	17	14	14	16	7.1%	-0.52 [-1.21, 0.18]
Rasmussen-Barr 2009	9.6	11	36	17	13	35	8.4%	-0.61 [-1.09, -0.13]
**Total (95% CI)**			**577**			**560**	**100.0%**	**-0.44 [-0.80, -0.09]**

Heterogeneity: Tau² = 0.33; Chi² = 88.75, df = 11 (P < 0.00001); I² = 88%
Test for overall effect: Z = 2.47 (P = 0.01)

Figure 2: Pooled main effect size estimates (standardized mean differences) for the outcomes pain intensity (-A-) and disability (-B-). Overall sustainability effects of motor control stabilisation exercise in comparison to inactive control, passive treatment or other exercises. MCE, motor control stabilisation exercise, SD, standard deviation; CI, confidence interval.

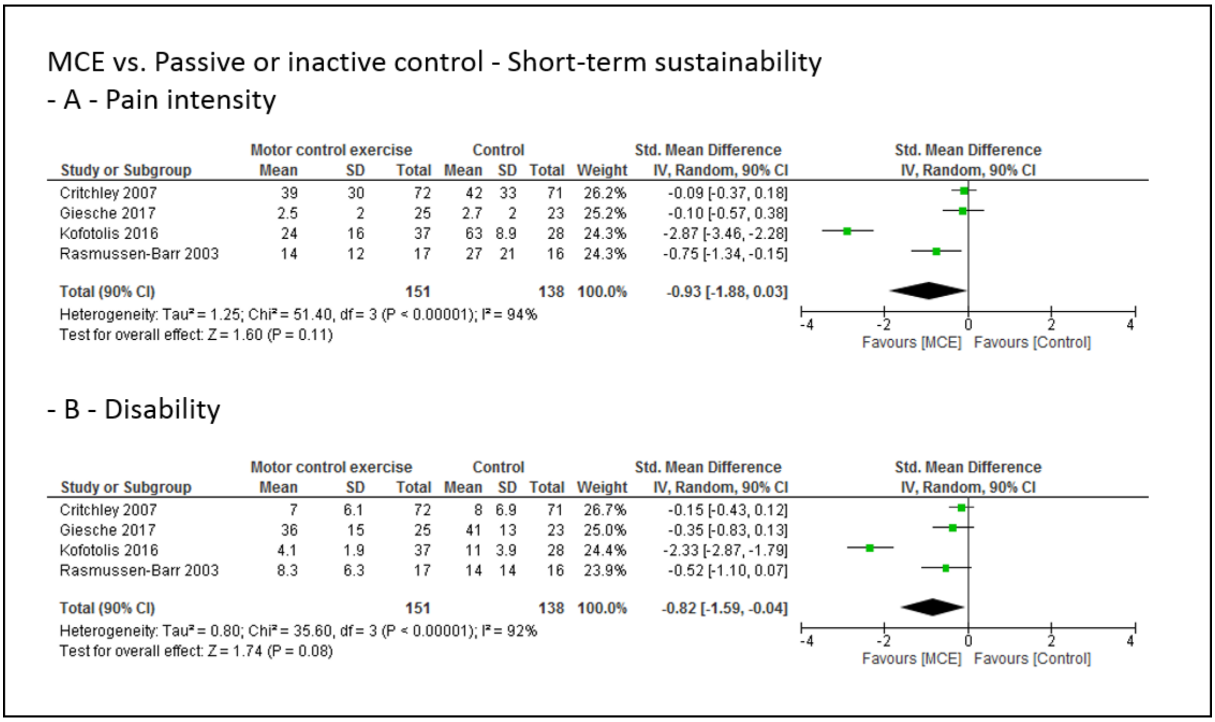

Figure 3: Pooled effect sizes (standardized mean differences) for the outcomes pain intensity (-A-) and disability (-B-). Analysis for the short-term sustainability effects of motor control stabilisation exercise in comparison to passive or inactive control. MCE, motor control stabilisation exercise; SD, standard deviation; CI, confidence interval.

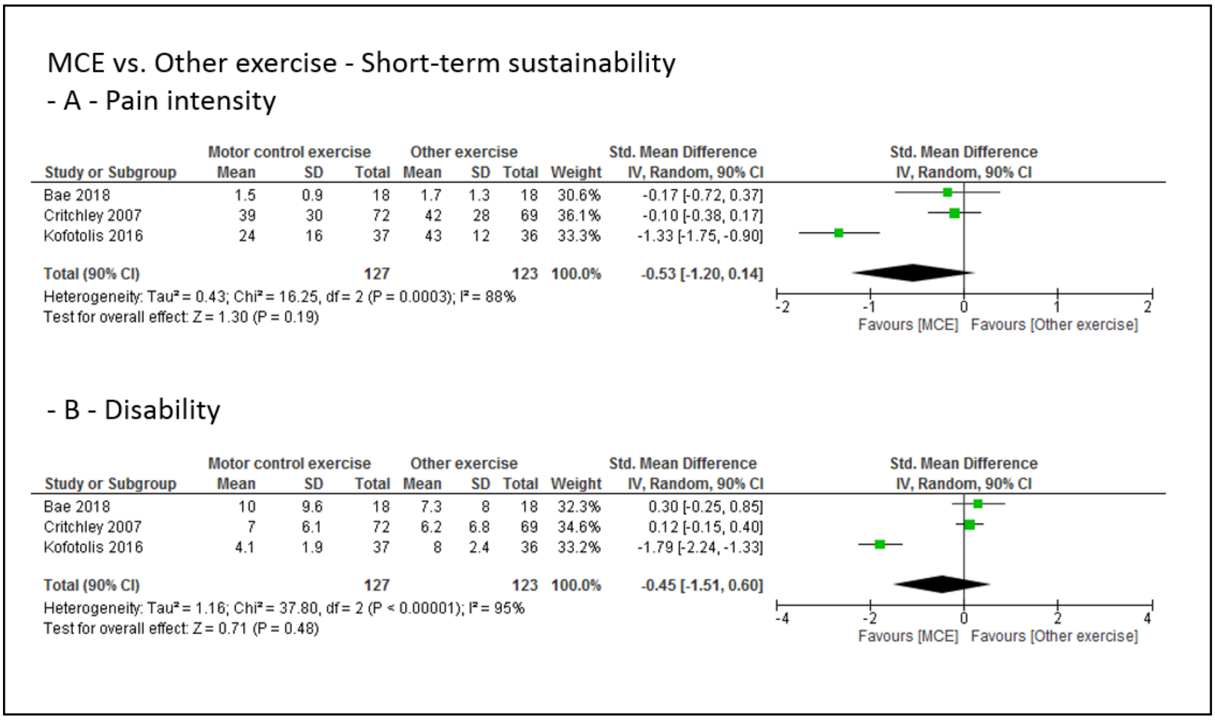

Figure 4: Pooled effect sizes (standardized mean differences) for the outcomes pain intensity (-A-) and disability (-B-). Analysis for the short-term sustainability effects of motor control stabilisation exercise in comparison to other exercises. MCE, motor control stabilisation exercise; SD, standard deviation; CI, confidence interval.

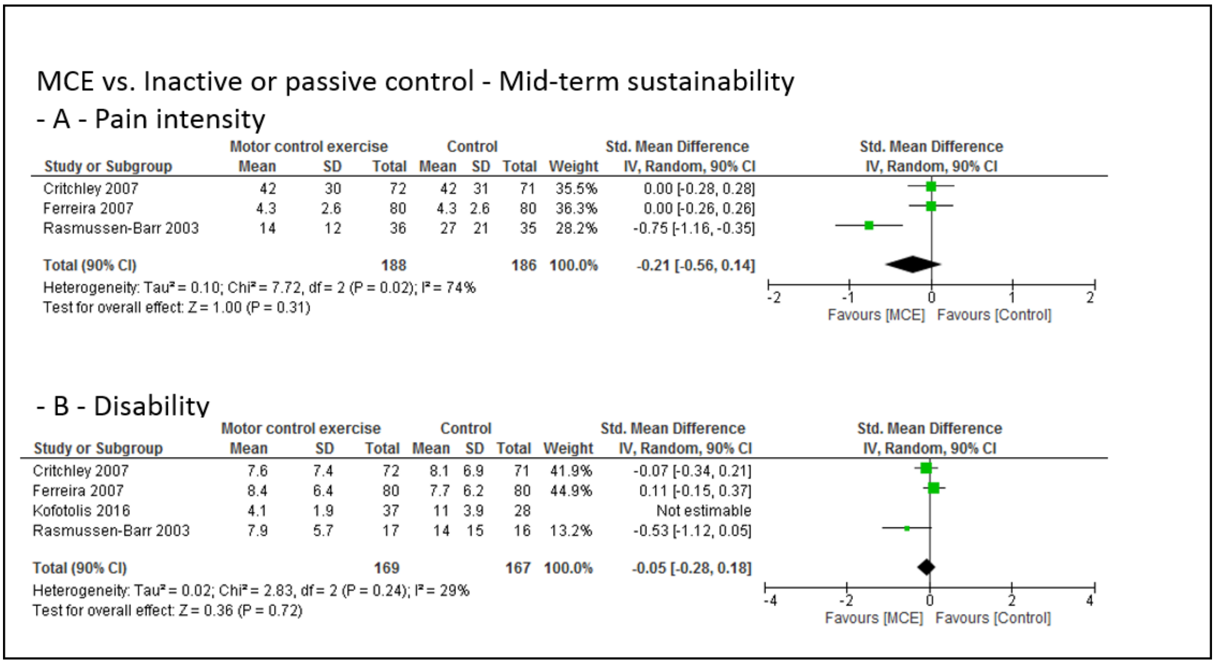

Figure 5: Pooled effect sizes (standardized mean differences) for the outcomes pain intensity (-A-) and disability (-B-). Analysis for the mid-term sustainability effects of motor control stabilisation exercise in comparison to passive or inactive control. MCE, motor control stabilisation exercise; SD, standard deviation; CI, confidence interval.

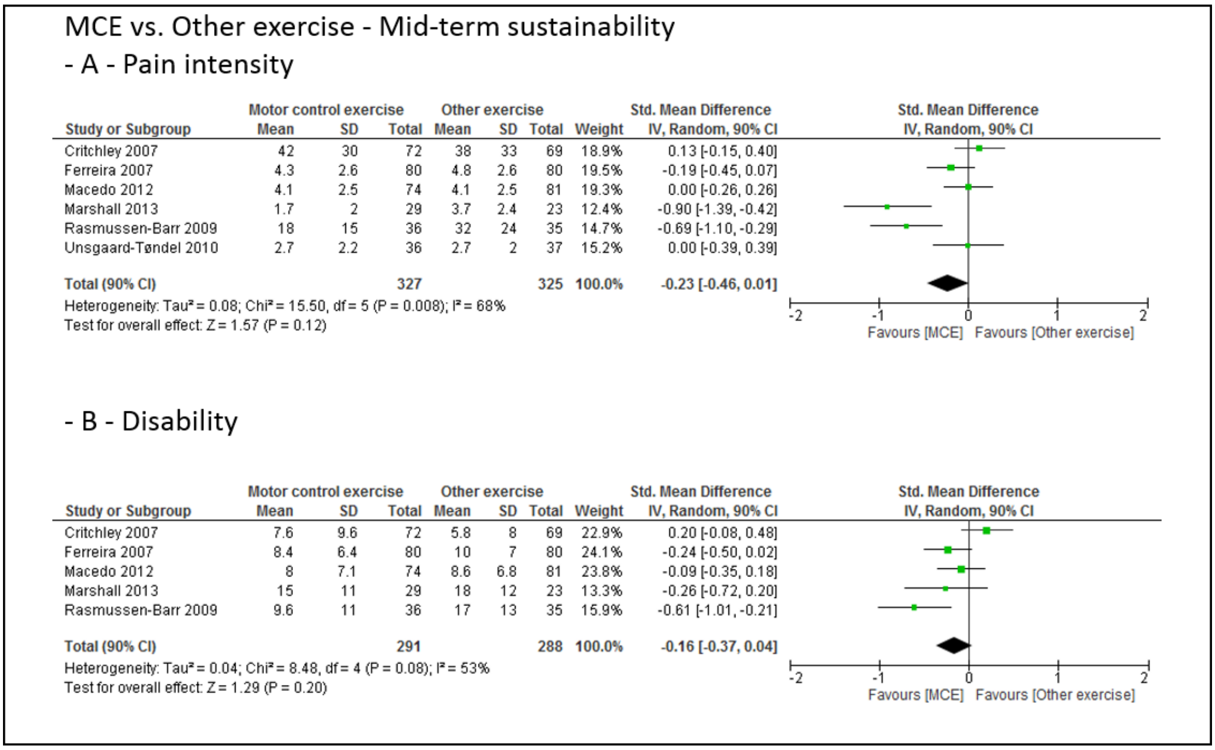

Figure 6: Pooled effect sizes (standardized mean differences) for the outcomes pain intensity (-A-) and disability (-B-). Analysis for the mid-term sustainability effects of motor control stabilisation exercise in comparison to other exercises. MCE, motor control stabilisation exercise; SD, standard deviation; CI, confidence interval.

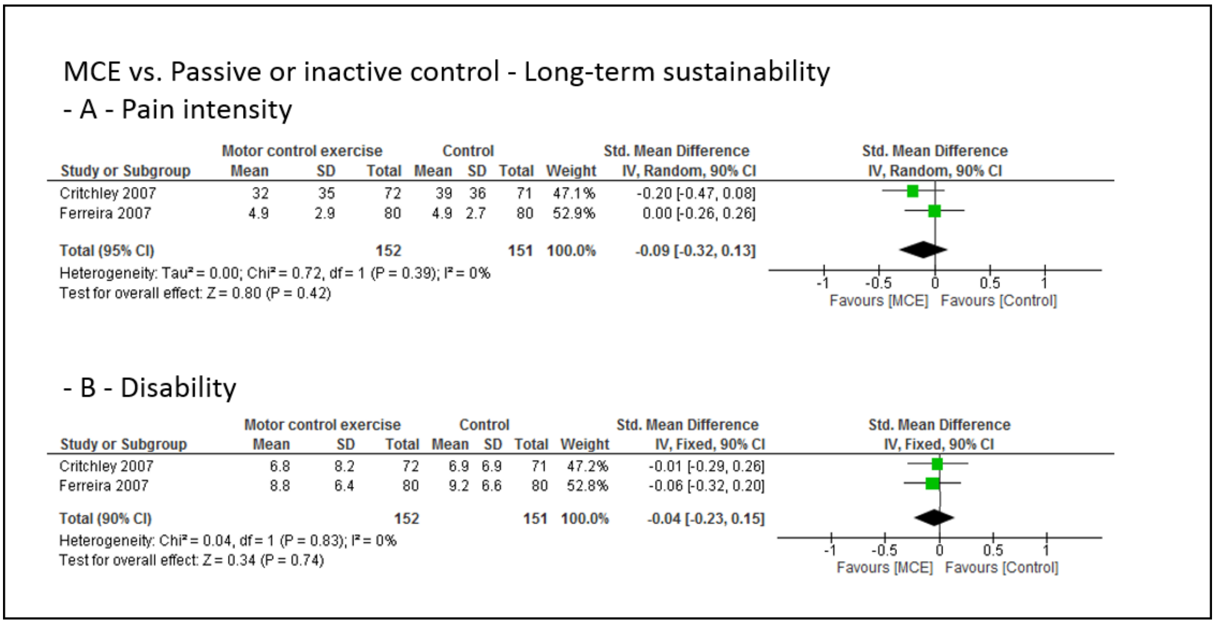

Figure 7: Pooled effect sizes (standardized mean differences) for the outcomes pain intensity (-A-) and disability (-B-). Analysis for the long-term sustainability effects of motor control stabilisation exercise in comparison to passive or inactive control. MCE, motor control stabilisation exercise; SD, standard deviation; CI, confidence interval.

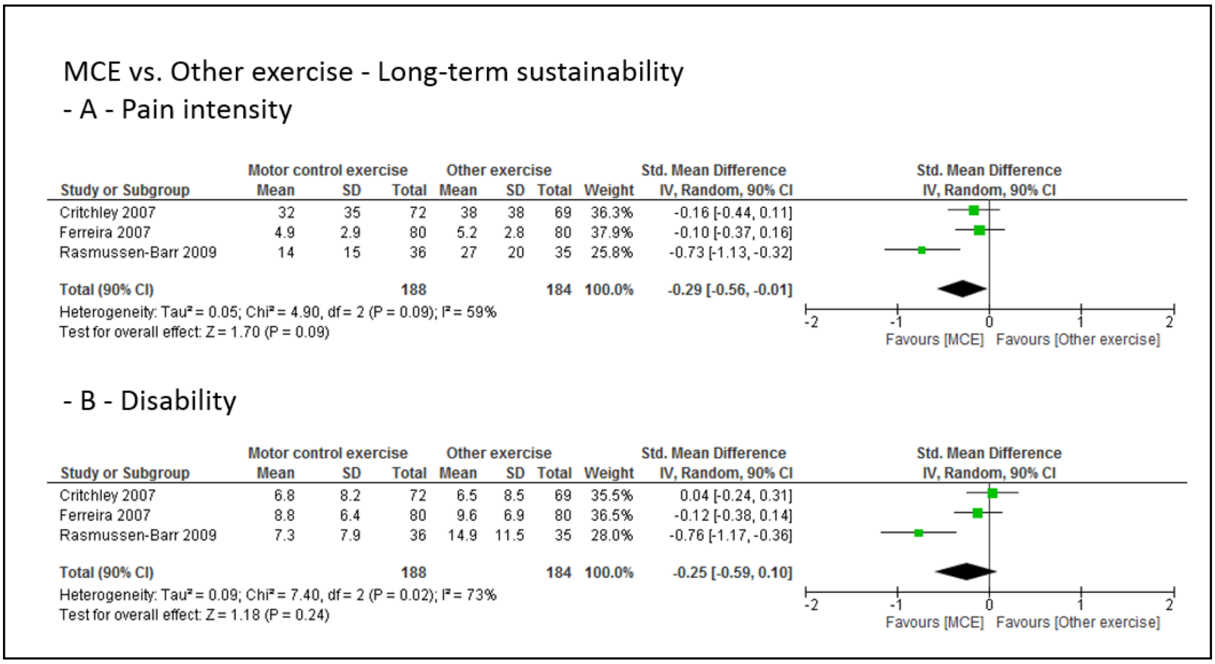

- A - Pain intensity

Study or Subgroup	Motor control exercise Mean	SD	Total	Other exercise Mean	SD	Total	Weight	Std. Mean Difference IV, Random, 90% CI
Critchley 2007	32	35	72	38	38	69	36.3%	-0.16 [-0.44, 0.11]
Ferreira 2007	4.9	2.9	80	5.2	2.8	80	37.9%	-0.10 [-0.37, 0.16]
Rasmussen-Barr 2009	14	15	36	27	20	35	25.8%	-0.73 [-1.13, -0.32]
**Total (90% CI)**			**188**			**184**	**100.0%**	**-0.29 [-0.56, -0.01]**

Heterogeneity: Tau² = 0.05; Chi² = 4.90, df = 2 (P = 0.09); I² = 59%
Test for overall effect: Z = 1.70 (P = 0.09)

- B - Disability

Study or Subgroup	Motor control exercise Mean	SD	Total	Other exercise Mean	SD	Total	Weight	Std. Mean Difference IV, Random, 90% CI
Critchley 2007	6.8	8.2	72	6.5	8.5	69	35.5%	0.04 [-0.24, 0.31]
Ferreira 2007	8.8	6.4	80	9.6	6.9	80	36.5%	-0.12 [-0.38, 0.14]
Rasmussen-Barr 2009	7.3	7.9	36	14.9	11.5	35	28.0%	-0.76 [-1.17, -0.36]
**Total (90% CI)**			**188**			**184**	**100.0%**	**-0.25 [-0.59, 0.10]**

Heterogeneity: Tau² = 0.09; Chi² = 7.40, df = 2 (P = 0.02); I² = 73%
Test for overall effect: Z = 1.18 (P = 0.24)

Figure 8: Pooled effect sizes (standardized mean differences) for the outcomes pain intensity (-A-) and disability (-B-). Analysis for the long-term sustainability effects of motor control stabilisation exercise in comparison to other exercises. MCE, motor control stabilisation exercise; SD, standard deviation; CI, confidence interval.

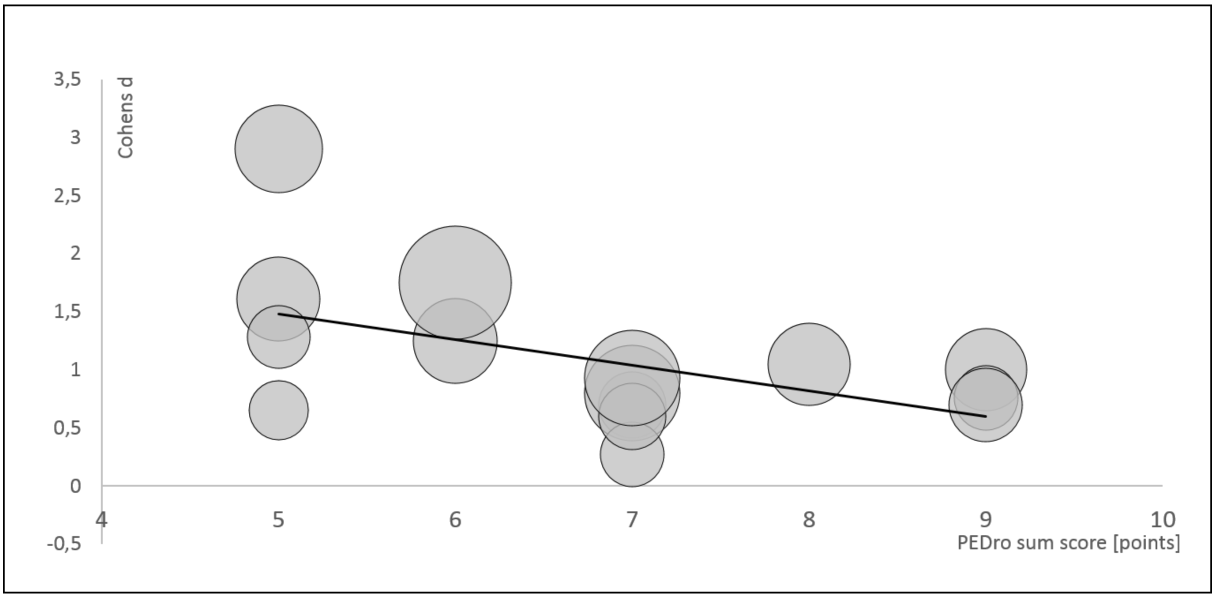

Figure 9: Meta-regression bubble plot for the dependent variable Cohens d, independent variable PEDro sum score and weighting (illustrated by the size of the bubbles).

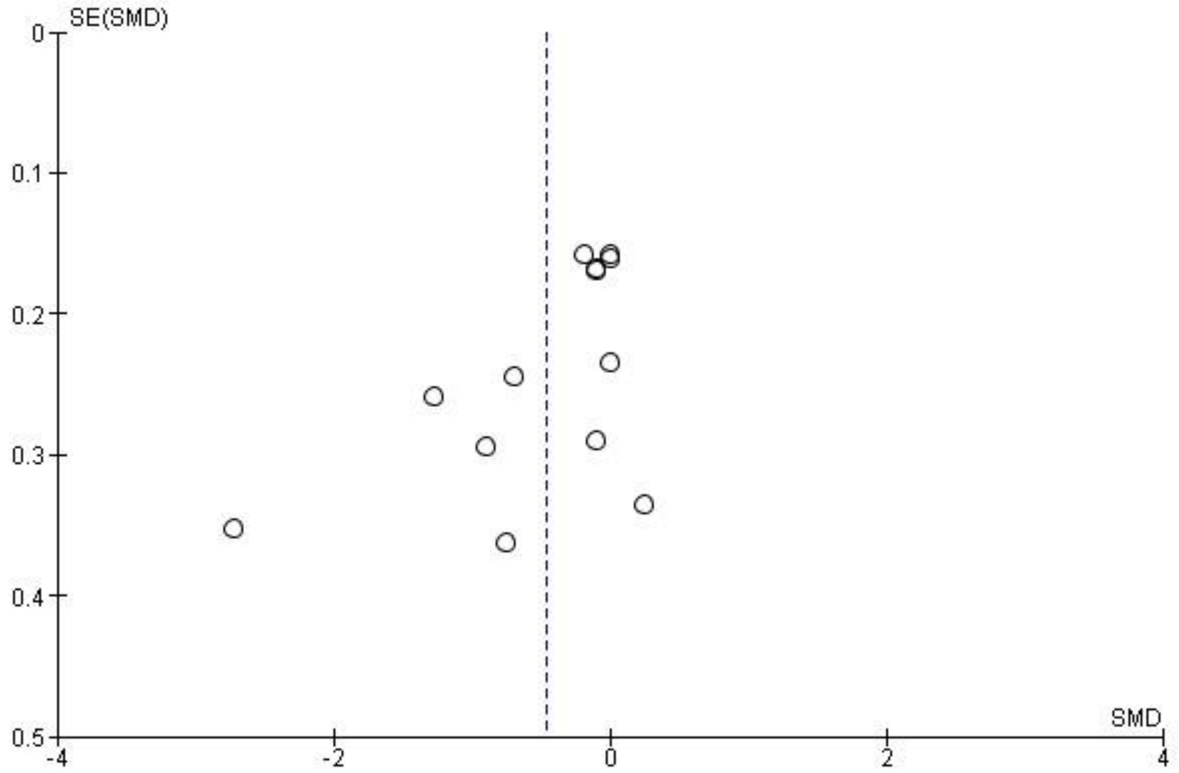

Figure 10: Funnel plot of all studies included. Each first sustainability SMD (standard mean differences and their belonging SE (standard errors) are plotted

Checklist

Click here to access/download
Supporting Information
PRISMA checklist.doc
